# Settling the score: what composite measures of social determinants tell us about hypertension risk

**DOI:** 10.1093/jncics/pkae065

**Published:** 2024-09-02

**Authors:** William Letsou

**Affiliations:** Department of Biological and Chemical Sciences, New York Institute of Technology, Old Westbury, NY, USA

In the United States, an estimated 18 million or more individuals are living with cancer (1), among whom approximately 500 000 are survivors of childhood cancer (2). Cancer survivorship rates vary globally and by tumor site, but for many common malignancies in developed countries, 5-year survival is around 80% (3). Although much of this gain in survival has been because of the introduction of combination chemotherapy protocols in the 1960s and 1970s (4), progress has continued in the past several decades (2,3,5,6). Alongside these successes has come the need to recognize and mitigate the increased risk of treatment-related comorbidities that cancer survivors face relative to the general population (7).

Among the most prevalent cancer-related comorbidities is hypertension (7,8). Essential hypertension, typically defined as a blood pressure reading of at least 140/90 mm Hg, is a diagnosis of worldwide concern for cancer survivors and the general population alike (9). Elevated blood pressure is a risk factor of severe cardiac events, such as coronary artery disease and heart failure (10,11), and is known to be linked to modifiable risk factors such as obesity and smoking (12). Hypertension is more common in adult survivors of childhood cancer than in their cancer-free siblings or the general population (13-15), and attains a cumulative prevalence of 40% to 70% in this group by age 50 years (vs 25%-40% in individuals without cancer) (13,16). Moreover, the presence of hypertension, combined with treatment-related risk factors such as anthracyclines and chest-directed radiation therapy, can dramatically increase the risk ratios of major cardiac events in childhood cancer survivors (16). Thus, it is especially important for physicians to be aware of the added risk hypertension plays in the cardiac health of cancer survivors.

The challenge for epidemiologists is to predict and stratify hypertension risk in this group of patients. Chemotherapy, including anthracyclines and alkylating agents, and radiation therapy have been shown to be associated with increased risk of hypertension (15,17) and other cardiac events (14,16,18), although the hypertension relationship has not always been borne out in large studies (13). Where anthracyclines are specifically associated with cardiomyopathy and heart failure by their generation of free radicals and alcohol intermediates as well as inhibition of topoisomerase IIβ in heart muscle (19-21), drugs that interfere with the vascular endothelial growth factor pathway and blood vessel endothelium are known to lead to hypertension (22-24), which in turn exacerbates anthracycline-induced cardiotoxicity in children and adults (16,25). Genetics likely play a role in the increased susceptibility to anthracycline-induced cardiotoxicity (18,26,27) and hypertension more broadly (28,29), but there is evidence that other factors, such as stress (30) and social determinants of health (SDOH) (31), are independently associated with worsened cardiovascular health among cancer survivors.

It is against this backdrop that Nain et al. (32) have conducted a survival analysis of hypertension risk in adult cancer survivors using a composite score of SDOH, the results of which appear in this issue of the Journal. The authors measured blood pressure in a prospective study of more than 300 cancer survivors using a combination of in-home and clinical readings over a median interval of 6 months per patient. The follow-up rate was extraordinarily high, with more than 90% of patients returning for at least 2 clinical visits. The outcome of interest was uncontrolled hypertension, as determined by an average of in-home and clinical blood pressure readings greater than 140/90 mm Hg, and the independent variable was an SDOH tally score, derived from administration of the Protocol for Responding to and Assessing Patients’ Assets, Risks, and Experiences (PRAPARE) Screening Tool (33) at the patient’s first visit. PRAPARE is the result of a collaboration among 4 large health organizations whose goal it was to standardize the collection of SDOH data and its integration into electronic health records (33). Although other studies have attempted to explain hypertension risk by composite SDOH measures (34-36), including recently in cancer survivors (31), the study by Nain et al. (32) is the first to explain hypertension risk in cancer survivors using the PRAPARE instrument.

These studies show consistently and convincingly that a composite measure of adverse SDOH variables spanning the domains of personal characteristics, family and home environment, financial and other resources, and social and emotional health can explain increased hypertension risk. In the report by Nain et al. (32), an SDOH tally score of at least 5 was associated with a statistically significant increase in risk of uncontrolled hypertension in urban residents, and a borderline statistically significant increase in adults at least sixty-five years-old, compared to a score of less than 5, even after adjustment for age and sex, modifiable hypertension risk factors, and cancer metastasis and treatment (32). Surprisingly, cancer medication was not statistically significantly associated with increased hypertension risk (with about 1 in 3 reporting hypertension among those on and off medication), a result which shows that SDOH can complicate the expected action of a drug—although there was a borderline statistically significant interaction of cancer medication and SDOH tally score. These results therefore appear to vindicate calls for so-called “polysocial risk scores” (37) that aggregate the effects of many different components of a patient’s social environment into an holistic measure.

Yet, at the same time, much could be lost in such an approach. One criticism of polygenic risk scores (which inspire the composite SDOH approach) is that an ability to stratify risk in the population does not necessarily translate into reliable estimates of individual risk, especially in the context of rare diseases (38,39), which hypertension is of course not. One source of this discrepancy is the fact that SDOH are multidimensional, and that collapsing them into a single score hazards masking and confounding potentially informative indicators. Consider, for example, that Nain et al. (32) find a statistically significant effect of SDOH tally score on hypertension risk in urban but not rural residents. Small sample size for the latter group notwithstanding, hypertension is known to be more prevalent in rural areas than in urban areas (40); the fact that SDOH do not modify rural risk further may lead one to hypothesize that geography overwhelms the effect of the composite score. We have also to consider complex scenarios such as the reverse-possibility of hypertension leading to poor SDOH scores, specifically in urban environments. Neither of these events can be effectively captured by a single score; rather, they require an analysis of coincidences of specific events that may be of more relevance to individual-level risk.

Can such specific indicators be found without complex study designs? Recent results suggest yes. A study of hypertension risk among Black, Latino, and White adults living in Los Angeles, California, found that although neighborhood socioeconomic disadvantage was in fact associated with a statistically significant increase in disease risk, higher levels of neighborhood organizational participation tended to decrease the Black-White risk disparity, an effect that was greatest among Black adults (41). This method of allowing the SDOH tally score to fall with mitigating positive factors is not possible if adverse risk factors are simply summed. Another study of the risk of cardiovascular disease in female breast cancer survivors found that residence in neighborhoods of relatively high Asian American and/or Pacific Islander composition was protective, but only if the neighborhoods were also of high socioeconomic status (42). Such information can be lost if different categories of SDOH are collapsed into a single score. As a final example, a report from the Jackson Heart Study found that the protective effects of social cohesion on the incidence of type 2 diabetes in African Americans was partially masked by the availability of “unfavorable” foods (43), suggesting that, particularly when SDOH affect risk in complex ways, composite scores that combine the effect of many counteracting variables threaten to conceal the risk-increasing or risk-decreasing effect associated with each.

It is understandable that when different studies reveal such disparate and, at times, conflicting and borderline statistically significant associations between individual SDOH and disease risk, we should like a summary statistic that tells us that the social environment in which we live can indeed affect health and well-being. These scores are probably best put to use as cues for closer follow-up of the variables they comprise. For although Nain et al. (32) have done a commendable job in showing the role of SDOH on hypertension risk in a particularly susceptible group, and have guarded against oversimplification of the complex social contributions to disease risk that comes of considering them in isolation, we should also not err in the opposite direction by oversimplifying the way in which they interact.

## Data Availability

No new data were generated or analyzed for this editorial.
